# Relationship between LTR Methylation and *gag* Expression of HIV-1 in Human Spermatozoa and Sperm-Derived Embryos

**DOI:** 10.1371/journal.pone.0054801

**Published:** 2013-01-28

**Authors:** FangZheng Li, LianBing Li, Ying Zhong, QingDong Xie, JiHua Huang, XiangJin Kang, Dian Wang, Lan Xu, TianHua Huang

**Affiliations:** 1 Guangdong Provincial Key Laboratory of Infectious Diseases and Molecular Immunopathology, Research Center for Reproductive Medicine, Shantou University Medical College, Shantou, Guangdong, China; 2 Key Laboratory of Birth Defects and Reproductive Health, Chongqing, China; 3 Center for Reproductive Medicine, Chengdu Jinjiang Hospital for Maternal and Child Health Care, Chengdu, Sichuan, China; National Institute of Health, United States of America

## Abstract

**Objective:**

Studying the methylation status of long terminal repeats (LTR) and its relationship to *gag* expression of HIV-1 in order to explore regulation mechanism of HIV-1 gene expression in vertical transmission from sperm to embryo.

**Methods/Principal Findings:**

Sperm samples were collected from a healthy donor and seven patients with HIV/AIDS. Zona-free hamster ova were fertilized by donor’s spermatozoa transfected with pIRES2-EGFP-LTR-*gag* and patient’s spermatozoa to obtain zygotes and 2-cell embryos, respectively. Interspecific *in vitro* fertilization, bisulfite sequencing PCR (BSP), RT-PCR, nested RT-PCR, nested real-time qRT-PCR and 2^−△△*C*t^ method, indirect immunofluoresence (IF) assay were performed. For donor’s samples, the methylation rates of HIV-1 LTR were 0.56%, 1.67%, 0.56%, 0.56% in plasmid, spermatozoa, zygotes and 2-cell embryos, respectively while spermatozoa were transfected with unmethylated plasmid, and were 95.0%, 84.44%, 3.3%, 1.67% while transfected with methylated plasmid. The positive bands for HIV-1 *gag* cDNA were detected in spermatozoa and 2-cell embryos. The positive signals for HIV-1 p24 Gag protein were detected in 2-cell embryos but not in spermatozoa. For patient’s samples, methylation rates of HIV-1 LTR were different in spermatozoa among patients. After fertilization, CpG sites in HIV-1 LTR were highly demethylated in zygotes and 2-cell embryos. The *gag* transcription levels increased with decreasing of methylation rates of HIV-1 LTR, which showed a strong negative correlations between *gag* transcription levels and methylation rates of HIV-LTR ether in the spermatozoa (r = −0.9877, P<0.0001) or in the sperm-derived 2-cell embryos (r = −0.9092, P = 0.0045).

**Conclusion:**

LTR methylation regulates expression of HIV-1 *gag* in vertical transmission from sperm to embryo.

## Introduction

Human immunodeficiency virus type 1 (HIV-1) infection is characterized by a prolonged period of latency, which may be followed by AIDS or AIDS-related complex [1]. The dramatic spread of HIV/AIDS worldwide represents a serious menace to human health, survival and social development. Understanding of the routes of HIV-1 transmission and its mechanism is an important prerequisite for controlling the HIV/AIDS pandemic. The typical routes of HIV-1 transmission are sexual intercourse, blood-to-blood contact, and perinatal transmission from mother-to-child [2]–[5]. In the recent years, the true vertical transmission of HIV-1 via germ line, a new but not yet fully proven route, has attracted plenty of attention. Some reported the presence of HIV-1 RNA, proviral DNA or protein in ejaculated spermatozoa or maturing spermatids from HIV-1 seropositive men [6]–[8]. Baccetti et al. observed that HIV-1 can bind to and enter normal sperm, and the viral particles, their nucleic acids and antigens were present in the cytoplasm of sperm from HIV-1 infected men, and such sperm can transfer HIV-1 like particles to normal human oocytes through *in vitro* fertilization [9]. Thereafter, it was reported that HIV-1 proviral DNA can integrate into the genomes of human sperm and mouse oocytes which were transfected with the plasmid pIRES2-EGFP-gag, respectively. The sperm- or oocyte-introduced HIV-1 gag genes retained their functions in replication, transcription and translation in the embryonic cells [10], [11]. More recently, Wang et al. found that HIV-1 proviral sequences were able to integrate into sperm nuclei and chromosomes of HIV/AIDS patients. After being brought into oocyte by fertilization, it can replicate with the replication of host genome and subsequently express its protein in embryo [12]. These findings indicated the possibility of true vertical transmission of HIV-1 via spermatozoa. However, the mechanism for regulation of HIV-1 gene expression in spermatozoa and embryonic cells is still largely unknown.

DNA methylation is a very important manner not only for cells to regulate the expression of host genes but also to regulate the expression of viral genes entering into cells. In mammals there are at least two developmental periods, in germ cells and in preimplantation embryos, in which methylation patterns are reprogrammed genome wide [13]. In the male germ line, methylation acquisition begins in prospermatogonia and persists throughout spermatogenesis [14]–[17]. After fertilization, the active demethylation of paternal genome in zygote occurs before DNA replication followed by passive demethylation during cleavage stage, and de novo methylation was thought to happen after implantation [18]–[22]. CpG methylation of retroviral promoter and enhancer sequences situated in the 5′ LTR is considered to be a mechanism of transcriptional suppression [23]. HIV-1 can infect male germ cells and present in all developmental stages of spermatogenesis [6]–[8], [24], and be brought into embryo by fertilization [9], [10], [12]. These events just occur in the periods during which methylation patterns of host genome are reprogrammed genome-wide. So the following questions arouse our great interest: what changes in methylation status of HIV-1 LTR take place when virus enters spermatozoa? What changes in LTR methylation status of sperm-introduced HIV-1 genes take place in zygote and embryo after fertilization? What are the relationships between LTR methylation status and gene expression of HIV-1 in spermatozoa and sperm-derived embryos? In the present study, these questions were investigated to explore the regulation mechanism of HIV-1 *gag* expression in vertical transmission from sperm to embryo.

## Results

### Construction and Identification of the Plasmid

*The* successful *construction of the plasmid* pIRES2-EGFP-LTR-*gag* (in the text referred to shortly as “the plasmid”) *was* confirmed by *Vsp*I and *EcoR*I digestion, PCR amplification and DNA sequencing (data not show). A 2292-bp fragment was obtained by *Vsp*I and *EcoR*I digestion, and amplified from the constructed plasmid using a primer pair specific for LTR-*gag*, which identity with that amplified from the backbone plasmid (pSG3△env) was 99.64%, indicating that the fragment of HIV-1 LTR-*gag* DNA has been successfully cloned into the constructed plasmid.

### The Spermatozoa Transfected with the Unmethylated Plasmid

**Figure 1:**
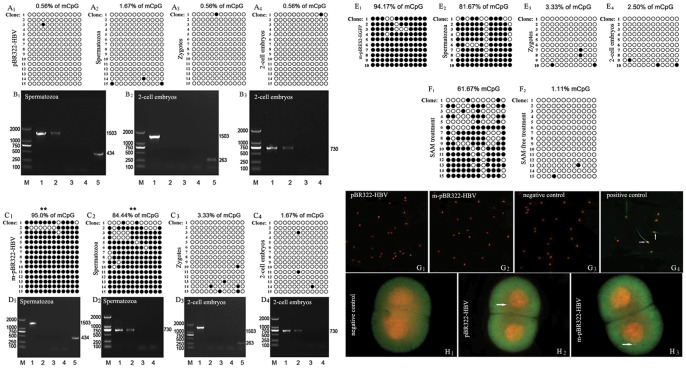
LTR methylation and gag expression of HIV-1 in the plasmid-transfected spermatozoa and sperm-derived zygotes and 2-cell embryos. A: The detection of LTR methylation by BSP and Thymine/Adenine cloning while transfection with unmethylated plasmid. The methylation status of 15 clones for each sample is presented; each column represents one CpG position in the U3-R region, with each circle in the column indicating either cytosine (open circles) or methyl cytosine (filled circles), the same below. (A_1_) plasmid; (A_2_) spermatozoa; (A3) zygotes; (A_4_) 2-cell embryos. B: The detection of *gag* transcription while transfection with unmethylated plasmid (B_1_) the results of RT-PCR. M: Marker; 1: positive control; 2: spermatozoa; 3: -RT; 4: -T; 5: human β-actin. (B_2_) the results of first-round of nested RT-PCR. M: Marker; 1: positive control; 2∶2-cell embryos; 3: -RT; 4: -T; 5: hamster β-actin. (B_3_) the results of second-round of nested RT-PCR. M: Marker; 1: positive control; 2: the first round product; 3: -RT; 4: -T. The results showed a clear correlation between LTR methylation and *gag* transcription of HIV-1 either in spermatozoa or in 2-cell embryos. C: The detection of LTR methylation while transfection with methylated plasmid. (C_1_) plasmid; (C_2_) spermatozoa; (C_3_) zygotes; (C_4_) 2-cell embryos. D: The detection of *gag* transcription while transfection with methylated plasmid. (D_1_) the results of first-round of nested RT-PCR. M: Marker; 1: positive control; 2: spermatozoa; 3: -RT; 4: -T; 5: human β-actin. (D_2_) The results of second-round. M: Marker; 1: positive control; 2: the first round product; 3: -RT; 4: -T. (D_3_) the results of first-round of nested RT-PCR M: Marker; 1: positive control; 2∶2-cell embryos; 3: -RT; 4: -T; 5: hamster β-actin. (D_4_) the results of second-round. M: Marker; 1: positive control; 2: the first-round product; 3: -RT; 4: -T. The results showed a clear correlation between LTR methylation and *gag* transcription of HIV-1 either in spermatozoa or in 2-cell embryos. E: CMV methylation in the spermatozoa transfected with methylated pIRES2-EGFP and sperm-derived zygotes and 2-cell embryos. The detection of CpG methylation by BSP and Thymine/Adenine cloning. (E_1_) m-pIRES2-EGFP; (E_2_) spermatozoa; (E3) zygotes; (E4) 2-cell embryos. The results showed that demethylation of some CpG sites in CMV promoter has already occurred in the spermatozoa and almost all CpG sites were demethylated in zygotes and 2-cell embryos. F: The effects of SAM on methylation of CpG sites in HIV-1 LTR in the 2-cell embryos. (F_1_) SAM treatment; (F_2_) SAM-free treatment. The results showed that the methylation rate of HIV-1 LTR in 2-cell embryos markedly increased after treatment with SAM. G: The detection of HIV-1 Gag translation in spermatozoa by IF assay. (G_1_) transfection with unmethylated plasmid; (G_2_) transfection with methylated plasmid; (G3) negative control; (G_4_) positive control. No positive signal for HIV-1 P24 Gag protein was observed in (G_1_), (G_2_) and (G_3_), and the strong signals for sperm protein were visible in (G_4_). H: The detection of Gag translation in 2-cell embryos by IF assay. (H_1_) negative control; (H_2_) derived from unmethylated sperm; (H_3_) derived from methylated sperm. The positive signal for HIV-1 P24 Gag protein was observed in (H_2_) and (H_3_). The results showed that HIV-1 *gag* was able to express its protein in the 2-cell embryos and not in the spermatozoa.

The methylation statuses of HIV-1 LTR in the unmethylated plasmid, sperm, zygote and 2-cell embryo were hypomethylated, and their methylation rates were 0.56%, 1.67%, 0.56% and 0.56%, respectively (Fig. 1 A1–A4).

By using RT-PCR, a 1503 bp positive band for HIV-1 *gag* cDNA was observed in the spermatozoa and positive control (unmethylated plasmid), and no positive band was detected in the negative controls (-RT, -T) (Fig. 1 B1). In the first round of nested RT-PCR, a 1503 bp positive band for HIV-1 *gag* cDNA was observed only in the positive control (Fig. 1 B2). In the second round of nested RT-PCR, the product of the first round was used as template, and a 730 bp positive band for HIV-1 P24 *gag* cDNA was observed in the 2-cell embryos and positive control, and no positive band was detected in the negative controls (-RT, -T) (Fig. 1 B3). The results showed that HIV-1 *gag* mRNA was present in the transfected spermatozoa and sperm-derived 2-cell embryos.

### The Spermatozoa Transfected with the Methylated Plasmid

The methylation status of HIV-1 LTR in the methylated plasmid was hypermethylated, and its methylation rate was 95.0% (Fig. 1 C1). In the transfected spermatozoa, some CpG sites in HIV-1 LTR began to be demethylated, and its methylation rate fell to 84.44% (Fig. 1 C2). In the zygote and 2-cell embryos, most CpG sites in HIV-1 LTR were demethylated, and their methylation rates were 3.33% and 1.67%, respectively (Fig. 1 C3 and C4). As the control, the plasmid pIRES2-EGFP with CMV promoter was treated by bisulfite modification and analyzed. The methylation status of CMV promoter in the methylated pIRES2-EGFP was hypermethylated, and its methylation rate was 94.17% (Fig. 1 E1). In the transfected spermatozoa, some CpG sites in CMV promoter also began to be demethylated, and its methylation rate fell to 81.67% (Fig. 1 E2). In the zygotes and 2-cell embryos, most CpG sites in CMV promoter were demethylated, and their methylation rates were 3.33% and 2.5%, respectively (Fig. 1 E3 and E4).

In the first round of nested RT-PCR, a 1503 bp positive band for HIV-1 *gag* cDNA was observed only in the positive control (Fig. 1 D1 and D3). In the second round of nested RT-PCR, the product of the first round was used as template, and a 730 bp positive band for HIV-1 P24 *gag* cDNA was observed in the spermatozoa, 2-cell embryos and positive control, and not in the negative controls (-RT, -T) (Fig. 1 D2 and D4). The results showed that HIV-1 *gag* mRNA was present in the transfected spermatozoa and sperm-derived 2-cell embryos.

### The Transfection Efficiency

The transfection efficiencies of the unmethylated and methylated plasmids into the spermatozoa were 33.51±2.72% and 31.77±1.98%, respectively. There was no significant difference in transfection efficiency between two plasmids (P>0.05).

### Comparison of the Transcription level of HIV-1 *gag* Gene between the Spermatozoa Transfected with the Unmethylated and Methylated Plasmids and the Sperm-Derived Embryos

The methylation statuses of HIV-1 LTR in the spermatozoa transfected with the unmethylated and methylated plasmids were 1.67% and 84.44%, respectively (Fig. 1 A2 and C2). The result of real-time qRT-PCR showed that there are 10.24-fold changes in the transcription level of HIV-1 *gag* gene between spermatozoa transfected with the unmethylated and methylated plasmids, respectively (Table 1).

**Table 1:** Transcription of HIV-1 *gag* in the spermatozoa transfected with unmethylated and methylated plasmids, respectively, and in the sperm-derived 2-cell embryos.*

	Transcription of HIV-1 *gag* (2^−△△*Ct*^)	
Samples	Unmethylated	Methylated
Spermatozoa	10.24 (8.29–12.64)	1 (0.77–1.29)
2-cell embryos	1.04 (0.84–1.3)	1 (0.74–1.36)

* By using real-time quantitative RT-PCR and 2^−△△*C*t^ method, the data were presented as the fold change in gene expression normalized to an endogenous reference gene and relative to HIV-1 *gag* in the spermatozoa transfected with the methylated plasmid, and in the sperm-derived 2-cell embryos.

The methylation statuses of HIV-1 LTR in the 2-cell embryos derived from spermatozoa transfected with the unmethylated and methylated plasmids were 0.56% and 1.67%, respectively (Fig. 1 A4 and C4). The result of real-time qRT-PCR showed that there are only 1.04-fold changes in the transcription level of HIV-1 *gag* gene between two types of embryos (Table 1).

### Effects of SAM on LTR Methylation Status and *gag* Transcription of HIV-1

The methylation rate of HIV-1 LTR in SAM-treated 2-cell embryos was markedly increased (61.67%) than that in the untreated one (1.11%) (Fig. 1 F).

There were 0.49-fold changes in the expression level of HIV-1 *gag* between SAM-treated and untreated 2-cell embryos (Table 2).

**Table 2:** Transcription of HIV-1 *gag* in the 2-cell embryos treated and untreated with SAM.*

	Treatmentwith SAM	Untreatmentwith SAM
2^−△△CT^	0.49 (0.37–0.65)	1 (0.73–1.37)

* By using real-time quantitative RT-PCR and 2^−△△*C*t^ method, the data were presented as the fold change in gene expression normalized to an endogenous reference gene and relative to the untreated control.

### HIV-1 Gag Translation in the Transfected Spermatozoa and Sperm-derived Embryos

No signal for HIV-1 p24 Gag protein was observed in the spermatozoa transfected with the unmethylated and methylated plasmids and in the negative control. The strong signals for sperm protein were detected in the positive control (Fig. 1 G).

The positive signals for HIV-1 P24 Gag protein were detected in the 2-cell embryos derived from the spermatozoa transfected with the unmethylated and methylated plasmids, respectively. No positive signal for HIV-1 P24 Gag protein was detected in the negative control (Fig. 1 H).

### LTR Methylation Status and *gag* Transcription of HIV-1 in the Spermatozoa from Patients with HIV/AIDS and Sperm-derived Zygotes and 2-cell Embryos

**Figure 2:**
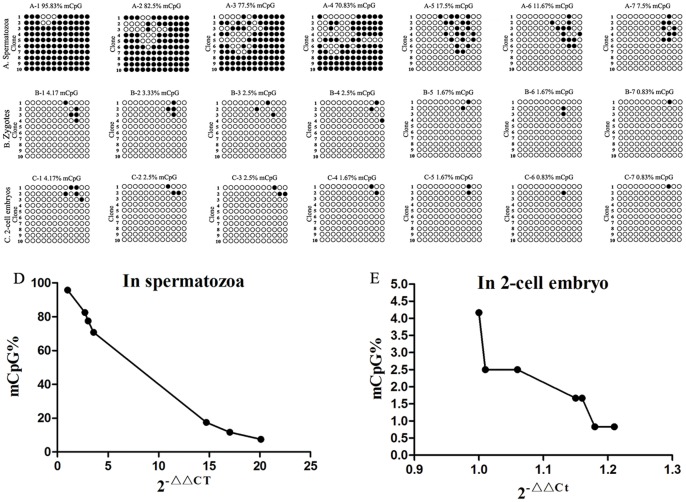
LTR methylation and *gag* expression of HIV-1 in the patient’s spermatozoa and sperm-derived 2-cell embryos. A1–A7: LTR methylation in the spermatozoa from seven patients with HIV/AIDS. B1–B7: in the zygotes derived from the patient’s spermatozoa. C1–C7: in the 2-cell embryos derived from the patient’s spermatozoa. The results showed that the methylation rates of HIV-1 LTR from the patient’s spermatozoa were different. After fertilization, the CpG sites in HIV-1 LTR were highly demethylated in the zygotes and 2-cell embryos, respectively. D: Correlation between LTR methylation rate (mCpG%) and *gag* transcription level (2^−△△*Ct*^) of HIV-1 in the patient’s spermatozoa. The *gag* transcription levels increased with decreasing of methylation rates of HIV-LTR. There were high negative correlations between *gag* transcription levels and methylation rates of HIV-LTR (Pearson r = −0.9877; P<0.0001). E: Correlation between LTR methylation rate (mCpG%) and *gag* transcription level (2^−△△*C*t^) of HIV-1 in the sperm-derived 2-cell embryos. The *gag* transcription levels increased with decreasing of methylation rates of HIV-LTR. There were high negative correlations between *gag* transcription levels and methylation rates of HIV-LTR (Pearson r = −0.9092; P = 0.0045).

To further confirm the correlation between LTR methylation statuses and *gag* transcription of HIV-1, the sperm samples were collected from seven patients with HIV/AIDS. The results showed in Table 3 and Figure 2.

**Table 3:** LTR methylation levels (mCpG%) and *gag* transcription of HIV-1 in the patient’s spermatozoa and sperm-derived zygotes and 2-cell embryos.*

Patients	Spermatozoa		Zygotes	2-cell embryos	
	mCpG%	2^−△△*C*t^	mCpG%	mCpG%	2^−△△*C*t^
1#	95.83	1.00	4.17	4.17	1.00
2#	82.50	2.71	3.33	2.50	1.01
3#	77.50	3.03	2.50	2.50	1.06
4#	70.83	3.58	2.50	1.67	1.15
5#	17.50	14.72	1.67	1.67	1.16
6#	11.67	17.03	1.67	0.83	1.18
7#	7.50	20.11	0.83	0.83	1.21

* By using real-time quantitative RT-PCR and 2^−△△*C*t^ method, the data on *gag* transcription were presented as the fold change in gene expression normalized to an endogenous reference gene and relative to *gag* in the spermatozoa from the patient with the highest level of LTR methylation, and in the sperm-derived 2-cell embryos, respectively.

The methylation rates of HIV-1 LTR in the spermatozoa from seven patients with HIV/AIDS were different, and the mean methylation rate was 51.9±37.03 (7.5% to 95.83%). After being brought into oocytes by fertilization, the CpG sites in HIV-1 LTR were highly demethylated and the mean methylation rates were 2.26±0.93 (0.83% to 4.17%) in the zygotes and 2.02±1.17 (0.83% to 4.17%) in the 2-cell embryos, respectively (Fig. 2 A–C; Table 3). There were significantly differences of the mean methylation rates of HIV-1 LTR between spermatozoa and sperm-derived zygotes and 2-cell embryos, respectively (P<0.05; P<0.05).

By using real-time qRT-PCR and 2^−△△*C*t^ method, the results showed that the *gag* transcription levels increased with decreasing of methylation rates of HIV-LTR in the spermatozoa and sperm-derived embryos (Fig. 2 D and E, Table 3). There were strong negative correlations between *gag* transcription levels and methylation rates of HIV-LTR ether in the spermatozoa (r = −0.9877, P<0.0001) or in the sperm-derived 2-cell embryos (r = −0.9092, P = 0.0045).

## Discussion

In the present study, the plasmid pIRES2-EGFP-LTR-*gag* contains both LTR regulatory elements and structural gene (*gag*) of HIV-1. Therefore, it could be used to directly and accurately investigate the relationship between LTR methylation and gene expression of HIV-1 *gag* transmitted from sperm to embryo. In addition, in our previous study, the spermatozoa from a healthy donor were co-incubated with the plasmid pIRES2-EGFP-*gag* [10], and the results showed that HIV-1 proviral sequences were able to integrate into sperm genome and sperm-introduced HIV-1 genes retained their function in replication and expression in the 2-cell embryos [10]. These results were the same as those in our recent study, in which the sperm samples were collected from the patients with HIV/AIDS [12]. Thus, in the present study the sperm samples from health donors, after being transfected with the plasmid, were used instead of sperm samples from patients with HIV/AIDS.

### The Methylation Status of HIV-1 LTR in the Spermatozoa and Sperm-derived Zygote and Embryo

In the spermatozoa transfected with the unmethylated plasmid, the methylation level of HIV-1 LTR increased little (1.67%) compared to that in the plasmid (0.56%). There was no significant difference in methylation rates between them (P>0.05). It suggested that there was no mechanism of de novo methylation of retroviral LTR in spermatozoa, which may be related to loss of methyltransferase (DNMT) activity because DNMT family members directly interact and cooperate to establish and maintain DNA methylation patterns in male germ cells, but establishment of methylation patterns occurs in spermatogonia and in spermatocytes not in spermatids [17], [25].

In the spermatozoa transfected with the methylated plasmid, the methylation level of HIV-1 LTR decreased (84.44%) compared to that in the plasmid (95.0%). As the control, in the spermatozoa transfected with the methylated pIRES2-EGFP, the methylation level of CMV promoter also decreased (81.67%) compared to that in the methylated pIRES2-EGFP (94.17%). These results indicated that there may exists a demethylation mechanism specific to some viral promoters including LTR of HIV-1 and CMV of cytomegalovirus not to host genome in spermatozoa because DNA demethylation of the paternal genomes began as early as 4–8 h after fertilization [26]–[29]. After being brought into oocytes by fertilization, most of CpG sites in LTR of HIV-1 and in CMV of cytomegalovirus were demethylated. For LTR of HIV-1, their methylation rates fell to 3.33% in the zygotes and 1.67% in the 2-cell embryos from 84.44% in the spermatozoa. For CMV of cytomegalovirus, their methylation rates fell to 3.33% in the zygotes and 2.50% in the 2-cell embryos from 81.67% in the spermatozoa.

In the spermatozoa from the patients with HIV/AIDS, the methylation levels of HIV-1 LTR were different in the range from 7.5% to 95.83%. After being brought into oocytes by fertilization, the CpG sites in HIV-1 LTR were also highly demethylated. Their mean methylation rates fell to 2.26±0.93 in the zygotes and 2.02±1.17 in the 2-cell embryos from 51.9±37.03 in the spermatozoa. There were significantly differences of the mean methylation rates of HIV-1 LTR between spermatozoa and sperm-derived zygotes and 2-cell embryos, respectively (P<0.05; P<0.05).

For host genome, the preferential demethylation of the paternal DNA in zygotes is a general phenomenon. Some reported that the components of the elongator complex (Elp1, Elp3 and Elp4) are important for paternal DNA demethylation [20], and reprogramming of the paternal genome upon fertilization involves genome-wide oxidation of 5-methylcytosine (5mC), and cytidine deaminases work in conjunction with DNA glycosylases to remove 5mC in a DNA repair pathway [26], [30]. Whether these factors also associate with demethylation mechanism of HIV-1 genes in spermatozoa, zygotes and preimplantation embryos? It should be explored further.

### Relationship between LTR Methylation and *gag* Expression of HIV-1 in the Spermatozoa and Sperm-derived Embryo

By using RT-PCR, a 1503 bp positive band for HIV-1 *gag* cDNA was detected in the spermatozoa transfected with the unmethylated plasmid but not in the spermatozoa transfected with the methylated plasmid, in which only a 730 bp positive band for HIV-1 P24 *gag* cDNA was observed by nested RT-PCR. When real-time qRT-PCR and the 2^−△△*C*t^ method were used to compare the transcription level of HIV-1 *gag* gene between the spermatozoa transfected with the unmethylated and methylated plasmids, respectively, the result showed that there were 10.24-fold changes in the transcription level of HIV-1 *gag* gene between two types of spermatozoa (Table 1). It suggested that the transcriptional level of HIV-1 *gag* was much lower in the methylated spermatozoa than in the unmethylated ones. In parallel, the methylation level of HIV-1 LTR in the methylated spermatozoa (84.44%) was much higher than in the unmethylated ones (1.67%), which suggested that the transcriptional level of HIV-1 *gag* decreased with increasing of the methylation level of HIV-1 LTR in the spermatozoa.

We collected the sperm sample from seven patients with HIV/AIDS and performed the real-time qRT-PCR and 2^−△△*Ct*^ method. The results also showed that the *gag* transcription levels increased with decreasing of the methylation level of HIV-1 LTR. There were strong negative correlations between *gag* transcription levels and methylation rates of HIV-LTR (r = −0.9877, P<0.0001).

Because of the limitation of embryo quantity used for the experiments, the nested RT-PCR was also applied to detect the transcription of HIV-1 *gag* in the 2-cell embryos. A 730 bp positive band for HIV-1 P24 *gag* cDNA was detected in the 2-cell embryos derived from the spermatozoa transfected with either unmethylated or methylated plasmid. When real-time qRT-PCR and 2^−△△Ct^ method were used to compare the transcription level of HIV-1 *gag* gene between both types of embryos, the results showed that the transcription levels of HIV-1 *gag* gene were very similar and only 1.04-fold changes (Table 1). In parallel, the HIV-1 LTR in both types of embryos were hypomethylated, and their methylation rates were 0.56% and 1.67%, respectively, which suggested that the transcription of HIV-1 *gag* was related to the methylation status of HIV-1 LTR. SAM is the methyl donor of numerous methylation reactions and can inhibit active demethylation [31]. The methylation rate of HIV-1 LTR in the 2-cell embryos treated with SAM markedly increased (61.67%), as compared with that in untreated embryos (1.11%). There were 0.49-fold changes in the expression level of HIV-1 *gag* between SAM-treated and untreated embryos, which suggested that the demethylation of HIV-1 LTR was synchronized with active demethylation of host genes in the 2-cell embryos, and the transcriptional level of HIV-1 *gag* was inversely correlated with its LTR methylation level.

To further confirm correlation between *gag* transcription levels and LTR methylation rates of HIV-1, the 2-cell embryos derived spermatozoa from seven patients with HIV/AIDS were assessed by using the real-time qRT-PCR and 2^−△△*C*t^ method. The results also showed that the *gag* transcription levels increased with decreasing of the methylation level of HIV-1 LTR. There were strong negative correlations between *gag* transcription levels and LTR methylation rates of HIV-1 (r = −0.9092, P = 0.0045<0.01).

No positive signal for HIV-1 Gag protein was detected in the spermatozoa transfected with either unmethylated or methylated plasmid. It suggested that HIV-1 *gag* was not able to express its protein in the spermatozoa, which is consistent with the observation in our previous *in vitro* study [10], but different from the detection in our *in vivo* study [12]. During the process of spermatogenesis, the spermatids mature into spermatozoa and lose most of their cytoplasm, leading to insufficient quantities of cytoplasmic ribosomes to support cytoplasmic mRNA translation [32]. Although spermatozoa were capable of using mRNAs transcripts for protein translation during the final maturation steps before fertilization, this nuclear-encoded mRNAs are translated by mitochondrial-type ribosomes not by the cytoplasmic translation machinery [33]. HIV-1 *gag* encodes the capsid proteins and its precursor (p55) is synthesized on cytoplasmic ribosomes not on mitochondrial-type ribosomes [34]. That is why our *in vitro* studies fail to detect the expression of Gag protein in the spermatozoa. HIV may infect germ cells early in spermatogenesis, resulting in the clonal transmission of the virus into spermatozoa [24]. In our *in vivo* study, the spermatozoa were collected from the patients with HIV/AIDS. If their germ cells were infected before losing cytoplasm, the cytoplasmic ribosomes are sufficient to support synthesis of *gag* precursor. This is the reason for detection of Gag protein expression in the spermatozoa.

After fertilization, oocytes are rich in cytoplasm which contains cytoplasmic ribosomes in sufficient quantities to support translation of host genes and sperm-introduced viral genes. Thus the positive signals for HIV-1 p24 Gag protein were observed in the 2-cell embryos derived from the sperm transfected with both types of plasmids, which suggested that the HIV-1 *gag* can express its protein in the 2-cell embryos.

Taken together, our data provide the solid evidences that LTR methylation regulates the expression of HIV-1 gag in the vertical transmission from sperm to embryo.

## Materials and Methods

### Ethical Approval

Mature 6–8-week old female hamsters were maintained under standard laboratory conditions (12 h light: 12 h darkness cycle). Human sperm samples were collected from a healthy male donor and seven patients with HIV/AIDS after written informed consents were received. All protocols used in the present study were approved by the Institutional Ethical Review Board (IERB) of Shantou University Medical College and conformed to the National Institutes of Health guidelines for humane animal care and use in research, and to the Ethical Principles for medical research involving human subjects of the 2008 WMA Declaration of Helsinki.

### Sperm Preparation

Human motile sperm were prepared as described previously [35], [36].

### Oocyte Preparation

The zona-free hamster ova were prepared as described previously [35], [36].

### Insemination and Post-insemination Culture

Insemination and post-insemination culture were performed as described previously [35], [36], in which the inseminated oocytes were divided into two groups. One group was incubated for 8 h to collect zygotes, and another group was incubated for 24 h until formation of 2-cell embryos.

### Construction and Identification of the Plasmid pIRES2-EGFP-LTR *-gag*

The cytomegalovirus (CMV) promoter of plasmid pIRES2-EGFP was replaced by LTR of HIV-1. The full length of LTR-*gag* DNA was amplified from the backbone plasmid pSG3Δenv (GenBank No. L02317) and subcloned into *Vsp* I and *EcoR* I sites of pIRES2-EGFP to generate the plasmid pIRES2-EGFP-LTR-*gag*. One pair of primers was used: forward, 5′- CGCTATTAATTGGAAGGGCTAATTCACT-3) with *Vsp* I restriction site in the 5′ region, and reverse, 5′- CCGGAATTCTTATTGTGACGAGGGGTCG-3′ with *Eco*R I restriction site in the 5′ region (Shanghai GeneCore Biotechnology Co., Ltd., Shanghai, China). The successful cloning was confirmed by PCR amplification, followed by restriction digestion mapping using *Vsp* I and *Eco*R I, and by DNA sequencing.

### Methylation of the Recombinant Plasmids

In the present study, the plasmid pIRES2-EGFP with CMV promoter was treated by bisulfite modification and analyzed as a control to assess the specificity of HIV LTR methylation. The methylation of the pIRES2-EGFP and pIRES2-EGFP-LTR-*gag* plasmids was performed in a 50 µl reaction containing the plasmid DNA (0.5 µg/µl, 5 µl), 10× NEBuffer (5 µl) (New England BioLabs, Beijing, China), S-adenosylmethionine (SAM, 1.6 mM, 5 µl), *CpG methyltransferase* (M. SssI, 4 U/ul, 2 ul) (New England Biolabs) and H_2_O (33 ul). After incubation at 37°C for 1 h, the reaction was stopped by heating at 65°C for 20 min. The DNA was purified by phenol-chloroform extraction and ethanol precipitation.

### Exposure of Human Spermatozoa to the Plasmid

The donor’s spermatozoa were transfected with the unmethylated and methylated plasmids, respectively. Briefly, 100 µl transfection complex containing 2 µg plasmid and 3 µl of FuGENE® HD Transfection Reagent (Roche, Guangzhou, China) were made in sterile water and kept at room temperature for at least 15 min. The spermatozoa were collected and incubated in fresh BWW medium supplemented with 1.5% BSA for capacitation. One hour later, the plasmid (final concentration of 1.5 µg/ml) was added to capacitating medium. After exposure and another capacitation for 3 h, the spermatozoa were washed 5 times to remove the plasmids, and then used to fertilize zona-free hamster ova *in vitro* to obtain zygotes and 2-cell embryos.

### Detection of Transfection Efficiency of Plasmids into the Spermatozoa

To measure the transfection efficiency of the unmethylated and methylated plasmids into the spermatozoa, the Nick Translation Kit (Roche, Basel, Switzerland) and Fluorescein-12-dUTP (Thermo Fisher Scientific, Waltham, Massachusetts, USA) were employed. Two types of plasmids were labeled with fluorescein-12-dUTP by using the nick translation according to manufacturer's instructions, respectively. The transfected spermatozoa can be recognized and counted by their green fluorescence under fluorescence microscope.

### Detection of Methylation Status of CpG Sites in HIV-1 LTR

A Universal Genomic DNA Extraction Kit (TaKaRa, Dalian, China) was used to extract the DNA from the transfected spermatozoa, sperm-derived zygotes and 2-cell embryos according to the manufacturer’s protocol. The bisulfite conversion of DNA, including HIV-1 LTR-*gag* segments integrated into the sperm genome and introduced into the zygotes and 2-cell embryos by fertilization, was performed using DNA Methylation-Gold™ Kit (ZYMO, Beijing, China) following the manufacturer’s instructions.

For the first round of nested PCR, the premix solution contained bisulfite-modified DNA (2 µl), *Taq™* Hot Start (12.5 µl) (TaKaRa), forward and reverse primers (10 µM, 0.5 µl each) and dH_2_O (9.5 µl). The primer pair consisted of forward (PA) 5′-TGGCAGAACTA CACA CCAGG GCCAG GGATC-3′ (nt 80 to 109) and reverse (PC) 5′-CCAGTATTTGTCTACAGCTTCTGCTGTTTC-3′ (nt 940 to 969). The PCR settings were as follows: denaturation at 95°C for 3 min; 40 cycles at 94°C for 30 s, at 55°C for 30 s, at 72°C for 1 min and extend at 72°C for 5 min. For the second round of nested PCR, the premix solution contained the first round PCR product (2 µl), *Taq™* Hot Start (12.5 µl), forward and reverse primers (10 µM, 0.5 µl each) and dH_2_O (9.5 µl). The primer pair consisted of forward (PB) 5′- AGAGAAGATAGAAGAGGCCAATGAAGGAGA-3′ (nt 160 to 189) and reverse (PE) 5′-GCTCCCAGGCTCAGATCTGGTCTAACCAGA-3′ (nt 460 to 489). The PCR settings were the same as above except at 72°C for 30 s instead of at 72°C for 1 min. The amplified products were cloned into pMD18*®*-T vector (TaKaRa) and sequenced. Only PCR clones with at least 95% conversion of cytosines outside CpN dinucleotides (N = A, T, or C) were taken into account.

### Detection of HIV-1 *gag* Transcription by RT-PCR and Nested RT-PCR

Total RNAs from the spermatozoa and sperm-derived 2-cell embryos were extracted as described previously [10]. RT-PCR and nested RT-PCR were performed according to the protocol of RNA PCR Kit (AMV) version 3.0 (TaKaRa). The unmethylated plasmid DNA was used as the positive controls, and the negative controls included minus reverse transcriptase (-RT) and minus template (-T). Human β-actin and Syrian hamster β-actin served as the internal controls. In RT-PCR and the first round of nested RT-PCR, the specific primers for HIV-1 *gag* were forward 5′-ATGGGTGCGAGAGCGTC-3′ and reverse 5′-TTATTGTGACGAGGGGTCG-3′. In the second round of the nested PCR, the first round product was used as the template, and the specific primers for HIV-1 P24 *gag* were forward 5′-AGCCAAAATTACCCTATAGTGCAGA-3′; and reverse 5′-TTGTTACTT GGCTCATTGCTTCA-3′.

### Detection of HIV-1 *gag* Transcription by Real-time qRT-PCR and Nested Real-time qRT-PCR

Total RNAs of the donor’s and patient’s spermatozoa were extracted, respectively and cDNA synthesis was performed as described previously [10]. Quantitative real-time RT-PCR was performed on a Chromo 4 real-time PCR machine (Bio-Rad), cDNA as a template with the primer pair (QGag2-F: 5′-ATGTTTTCAGCATTATCAGAAGGAG-3 and QGag2-R: 5′- TTCCTCATTGATGGTCTCTTTTA-3) in a 25 µl reaction mixture containing cDNA (2 µl), SYBR® *Premix Ex Taq*™ (12.5 µl) (TaKaRa), forward and reverse primers (10 µM, 0.5 µl each) and H_2_O (9.5 µl). Real-time PCR settings were as follows: at 50°C for 2 min, then at 95°C for 5 min, followed by 45 cycles at 95°C for 15 s and at 60°C for 1 min. The data analysis in singleplex assay was performed using the Chromo4 Thermal Cycler and Opticon Monitor 3 software (Bio-Rad).

Two-cell embryos, derived from donor’s and patient’s spermatozoa, respectively, in 1.5 µl phosphate buffered saline (PBS) were transferred to the bottom of a 200 µl Eppendorf tube containing 8.5 µl lysis buffer [37] and then the RNAs were extracted and reversely transcribed. The first round of nested real-time RT-PCR was performed on a conventional PCR machine (Bio-Rad PTC200) (Bio-Rad Laboratories, Hercules, CA) to amplify a 221 bp fragments of *gag*, using the primer pair (QGag1-F: 5′-TCAGCCCAGAAGTAATACCCATGT-3′ and QGag1-R: 5′-TGCTATGTCAGTTCCCCTTGGTTCTCT-3′) in a 25 µl reaction mixture containing cDNA (5 µl), *Taq™* Hot Start (12.5 µl), forward and reverse primers (10 µM, 0.5 µl each) and dH_2_O (6.5 µl). The PCR settings were as follows: denaturation at 94°C for 5 min; 15 cycles at 94°C for 30 s, at 55°C for 30 s, at 72°C for 1 min and then 72°C for 5 min. The second round was performed on a Chromo 4 real-time PCR machine (Bio-Rad), using the first round product as a template with the primer pair (QGag2-F: 5′-ATGTTTTCAGCATTATCAGAAGGAG-3 and QGag2-R: 5′- TTCCTCATTGATGGTCTCTTTTA-3) in a 25 µl reaction mixture containing the first PCR product (2 µl), SYBR® *Premix Ex Taq*™ (12.5 µl) (TaKaRa), forward and reverse primers (10 µM, 0.5 µl each) and H_2_O (9.5 µl). Real-time PCR settings were as follows: at 50°C for 2 min, then at 95°C for 5 min, followed by 50 cycles at 95°C for 15 s and at 60°C for 1 min. The data analysis in singleplex assay was performed using the Chromo4 Thermal Cycler and Opticon Monitor 3 software (Bio-Rad).

### Treatment with s-adenosylmethionine (SAM)

After fertilization of oocytes by sperm transfected with the methylated plasmid, SAM (8 mM, 1 µl) was added to a droplet (50 µl each) of OCM containing the inseminated ova at the beginning of post-insemination culture. After 24 h incubation, the 2-cell embryos were collected to detect methylation statuses of HIV-1 LTR by using BSP and to assess HIV-1 *gag* expressions by using nested real-time quantitative RT-PCR and the 2^−△△*C*t^ method.

### Detection of HIV-1 *gag* Translation Using IF Assay

To detect the expression of HIV-1 P24 Gag protein in the spermatozoa transfected with the unmethylated and methylated plasmids and sperm-derived 2-cell embryos, IF assay was performed as described previously [10]. The spermatozoa without transfection served as negative control, whereas detection of sperm protein using anti-human sperm polyclonal antibody and FITC-conjugated rabbit immunoglobulin served as positive control.

### Statistical Analysis

Data were presented as mean values ± SD. A paired-samples T test was performed by using SPSS 16.0 program to determine whether there is a significant difference in average methylation rates between spermatozoa and sperm-derived zygotes and 2-cell embryos. Pearson correlation and graphing were performed by using GraphPad Prism 5 software to measure the association between *gag* transcription levels and LTR methylation rates of HIV-1 in spermatozoa and sperm-derived 2-cell embryos. P-value of less than 0.05 was considered to be significant.
