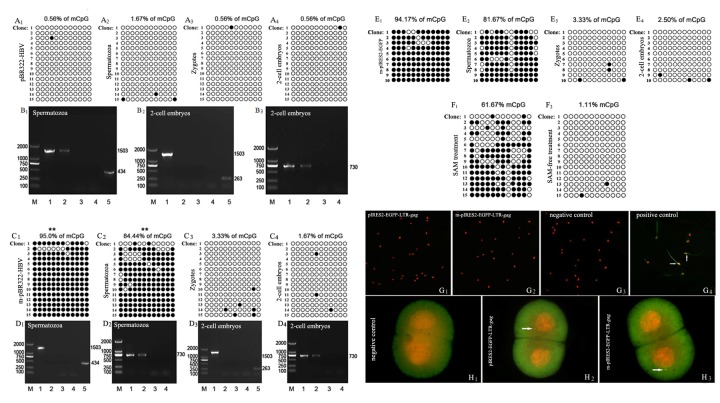# Correction: Relationship between LTR Methylation and *gag* Expression of HIV-1 in Human Spermatozoa and Sperm-Derived Embryos

**DOI:** 10.1371/annotation/021483ee-d8ff-4bc6-b780-b109f0054842

**Published:** 2013-12-09

**Authors:** FangZheng Li, LianBing Li, Ying Zhong, QingDong Xie, JiHua Huang, XiangJin Kang, Dian Wang, Lan Xu, TianHua Huang

The caption names for G_1_, G_2_, H_1_, H_3_ in Figure 1 are incorrect. Please see Figure 1 with the correct names here: 

**Figure:**